# Enhanced dissolution rate of felodipine using spherical agglomeration with Inutec SP1 by quasi emulsion solvent diffusion method

**Published:** 2009

**Authors:** A.R. Tapas, P.S. Kawtikwar, D.M. Sakarkar

**Affiliations:** 1 *Sudhakarrao Naik Institute of Pharmacy, Pusad-445204, Dist: Yavatmal, Maharashtra, India*; 2 Shri Sureshdada Jain Institute of Pharmaceutical Education and Research, Jamner-424206, Dist: Jalgaon, Maharashtra, India

**Keywords:** Felodipine, Spherical agglomeration, Inutec SP1, Dissolution rate, DSC, PXRD, FTIR

## Abstract

Felodipine is a second generation calcium channel blocker widely used as antihypertensive and antianginal drug which belongs to BCS class II category. Hence, its low water solubility limits the pharmacological effect. The aim of this study was to improve the dissolution rate of felodipine using spherical agglomeration technique with acetone, water and dichloromethane as good solvent, poor solvent and bridging liquid, respectively. The quasi emulsion solvent diffusion technique was used as a method for spherical agglomeration. Inutec SP1 was used as an emulsion stabilizer and as hydrophilic polymer in agglomeration process. The FTIR and DSC results showed no change in the drug after crystallization process. PXRD studies showed sharp peaks in the diffractograms of spherical agglomerates with minor reduction in height of the peaks. The particle size of spherical agglomerates (FI-2) was about 134.33 ± 13.57 µm, n=3 and the dissolution efficiency of felodipine up to 120 min increased to about 4-fold in phosphate buffer containing 1.8% Tween 80 (pH 6.8). Spherical agglomerates showed enhanced solubility compared to untreated powder possibly due to the partial conversion to amorphous form.

## INTRODUCTION

**Fig. 1:**
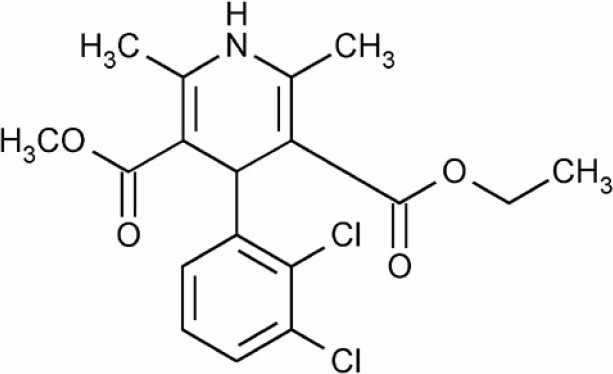
Chemical structure of FL

**Fig. 2:**
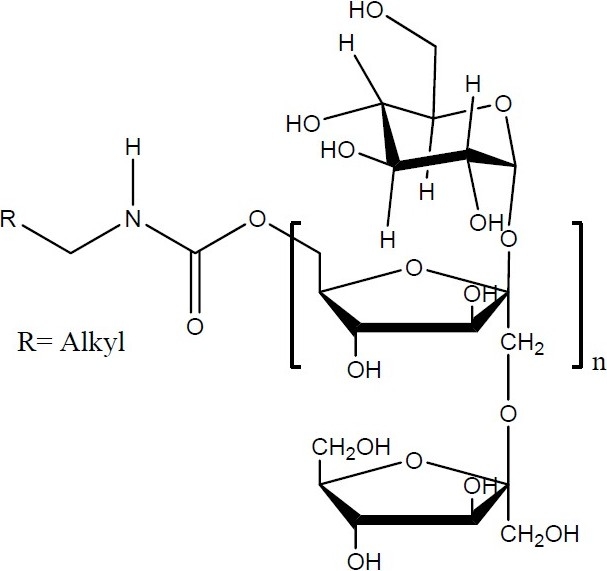
Chemical structure of Inutec SP1

Felodipine (FL) is (4RS)-4-(2,3-dichloro phenyl)-2,6-dimethyl-1,4-dihydropyridine-3,5-dicaboxylate (Fig. 1). It is a second generation calcium channel blocker widely used as anti-hypertensive and antianginal drug(1). According to biopharmaceutics classification system, felodippine is a class II drug i.e. low solubility and high permeability(2). FL has poor water solubility and hence poor dissolution and bioavailability after oral administration. It is extensively metabolized in the gut and the liver, and is excreted almost entirely as metabolites. About 70% of each dose is excreted in the urine; the remainder appears in the feces. Clinical studies have demonstrated that FL, which is approved for marketing in several countries, is an effective, well tolerated antihypertensive drug(3). Many technological methods regarding the enhancement of dissolution characteristic of drugs with low water solubility have been reported such as micronization, formation of solvates, adsorbates, complexes, microspheres, and solid dispersions(4). One of the approaches to enhance the dissolution rate is the use of spherical crystallization technique(5). Spherical crystallization has been developed by Yoshiaki Kawashima and co-workers as a novel particulate design technique to improve processibility such as mixing, filling, tableting characteristics and dissolution rate of pharmaceuticals(6). The resultant crystals can be designated as spherical agglomerates(7). This can be achieved by various methods such as spherical agglomeration, quasi emulsion solvent diffusion (QESD) and neutralization methods. Various polymers can be incorporated in the system to enhance the dissolution rate of drugs with poor dissolution profile(8). One such polymer is Inutec SP1 (Inulin Lauryl Carbamate). It is a derivative of inulin prepared by the reaction between isocyanates and the polyfructose backbone in the presence of a basic catalyst such as a tertiary amine or a Lewis acid(9). The structure of Inutec SP1 is shown in Fig. 2. In this way alkyl groups are introduced which are randomly distributed on the polysaccharide backbone. The resulting inulin carbamates possess tensioactive properties and can be used as an emulsifier in pharmaceutical formulations. It has been shown that in a two-phase system like an oilin-water emulsion, the alkyl groups become strongly adsorbed on the oil droplets, while the inulin chain is the stabilizing part of the molecule, forming loops dangling in solution(10). Other advantages of Inutec SP1 are a low viscosity and reservation of its stabilizing effect on emulsions and suspensions with high electrolyte concentrations. Hence the aim of present work was to enhance the dissolution rate of FL using spherical agglomeration with Inutec SP1 by QESD.

## MATERIALS AND METHODS

### 

#### Materials

FL USP was generously provided as a gift sample from Cipla Ltd., Mumbai Central, Mumbai, India. Inutec SP1 was obtained as a gift sample from Beneoorafti, Oreye, Belgium. Acetone and dichloromethane were purchased from Lobachemie, Mumbai, India. All other chemicals used were of analytical grade.

#### Preparation of agglomerates

All spherical agglomerates were obtained by QESD. Spherical agglomerates were prepared with and without Inutec SP1. The composition of Inutec SP1 is given in Table 1. FL (1.0 g) was dissolved in good solvent acetone (5.0 ml). The bridging liquid dichloro-methane (1.0 ml) was added to it. The resulting solution was then poured dropwise in to the poor solvent (distilled water, 75 ml) containing different concentrations of Inutec SP1 with a stirring rate of 800 rpm using propeller type agitator (Remi Motors Ltd., Mumbai, India) at room temperature. After agitating the system for 0.5 h, the prepared agglomerates were collected by filtration through whatmann filter paper no. 42, placed in a thin layer in an oven at 60 °C for 3 h.

**Table 1:** Composition of spherical agglomerates.

Ingredients	FL Agg	FI-1	FI-2
Felodipine (FL) (g)	1	1.0	1
Acetone (ml)	5	5	5
DCM (ml)	1	1	1
Inutec SP1 (% w/v)	0	0.5	1
Water (ml)	75	75	75
Stirring speed (rpm)	800	800	800

#### Fourier transform infrared spectroscopy (FTIR), differential scanning calorimetry (DSC) and Powder X-ray diffraction studies (PXRD)

The FTIR spectra of powder FL, and the agglomerates were recorded on an FTIR-spectrophotometer (IRAFFINITY-1, Shimadzu, Japan). DSC analysis was performed using a DSC 823 calorimeter (Mettler Toledo model) operated by STARe software. Samples of FL and its agglomerates were sealed in an aluminium crucible and heated at the rate of 10 °C/min up to 300 °C under a nitrogen atmosphere (40 ml/min). PXRD patterns of the pure drug and spherical agglomerates were monitored with an x-ray diffractometer (Panalytical Xpert pro MPD xrd machine) using copper as x-ray target, a voltage of 40 KV, a current of 30 mA and 1.5404 angstorm wavelength. Xcelerator RTMS with secondary monochromator was used as a detector. The samples were analyzed over 2θ range of 7.02--59.98° with scanning step size of 0.02° (2θ) and scan step time of one second.

#### Scanning electron microscopy (SEM)

The surface morphology of the agglomerates was accessed by SEM. The crystals were splutter coated with gold before scanning.

#### Percentage yield

The yield of spherical agglomerates was determined by comparing the whole weight of the agglomerates formed against the combined weight of the polymer and drug.

Eq. 1 $$Percentage\;Yield\;=\;\frac{Weight\;of\;agglomerates\;obtained}{Total\;weight\;of\;drug\;and\;Polymer\;used}\times 100$$

#### Drug loading efficiency

The drug loading efficiency of agglomerates was determined by dissolving 100 mg of crystals in 5 ml methanol and diluting further with distilled water (100 ml) containing 0.1% v/v Tween 80 as a wetting agent, followed by measuring the absorbance of appropriately diluted solution spectrophotometrically (PharmaSpec UV-1700, UV-Vis spectrophotometer, Shimadzu) at 362 nm.

#### Particle size analysis

The size of agglomerates was determined by microscopic method using stage and eyepiece micrometers. The shape of the agglomerates was observed under an optical microscope (×60 magnification) attached to a computer.

#### In vitro dissolution test

The *in vitro* dissolution studies were carried out using an 8 station USP 23 dissolution testing apparatus (Electrolab, India). The dissolution medium used was 900 ml of Phosphate buffer pH 6.8 containing 1.8% Tween 80(11–13). The dissolution medium was kept in a thermostatically controlled water bath at 37 ± 0.5 °C. A suitable amount of untreated powder or agglomerated crystals was dispersed in the dissolution medium. The medium was stirred at 75 rpm using paddle. The dissolution tests were carried out for 120 min. At predetermined time intervals, 5 ml of samples were withdrawn and analyzed spectrophotometrically. At each time of withdrawal, 5 ml of fresh corresponding medium was replaced into the dissolution flask. The cumulative amount of drug release was calculated and plotted versus time.

#### Dissolution efficiency studies

The dissolution efficiency (DE) of the batches was calculated by the method mentioned by Khan(14). It is defined as the area under the dissolution curve between time points t1 and t2 expressed as a percentage of the curve at maximum dissolution, y100, over the same time period or the area under the dissolution curve up to a certain time, t, (measured using trapezoidal rule) expressed as a percentage of the area of the rectangle described by 100% dissolution in the same time (Eq. 2)(15).

Eq. 2 $$\mathrm{Dissolution}\;\mathrm{efficiency}\;=\;\frac{\int_{0}^{}y\;dt}{y100\;\left(t_{2}\;-\;t_{1}\right)}\times \;100\%$$

DE_60_ values were calculated from dissolution data and used to evaluate the dissolution rate.

#### Statistical Analysis

The results obtained from the dissolution studies were statistically validated using ANOVA (Dunnett multiple comparisons test). DE was calculated using the origin software (Origin pro; version: 8.1).

## RESULTS

**Fig. 3:**
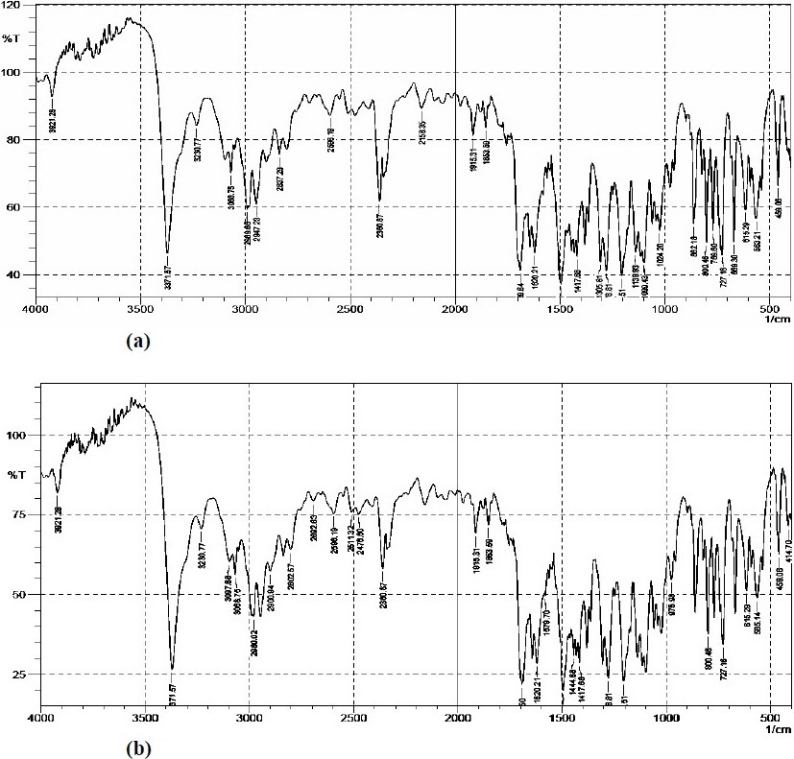
IR spectra of a) Felodipine b) Spherical agglomerates FI-2

**Fig. 4:**
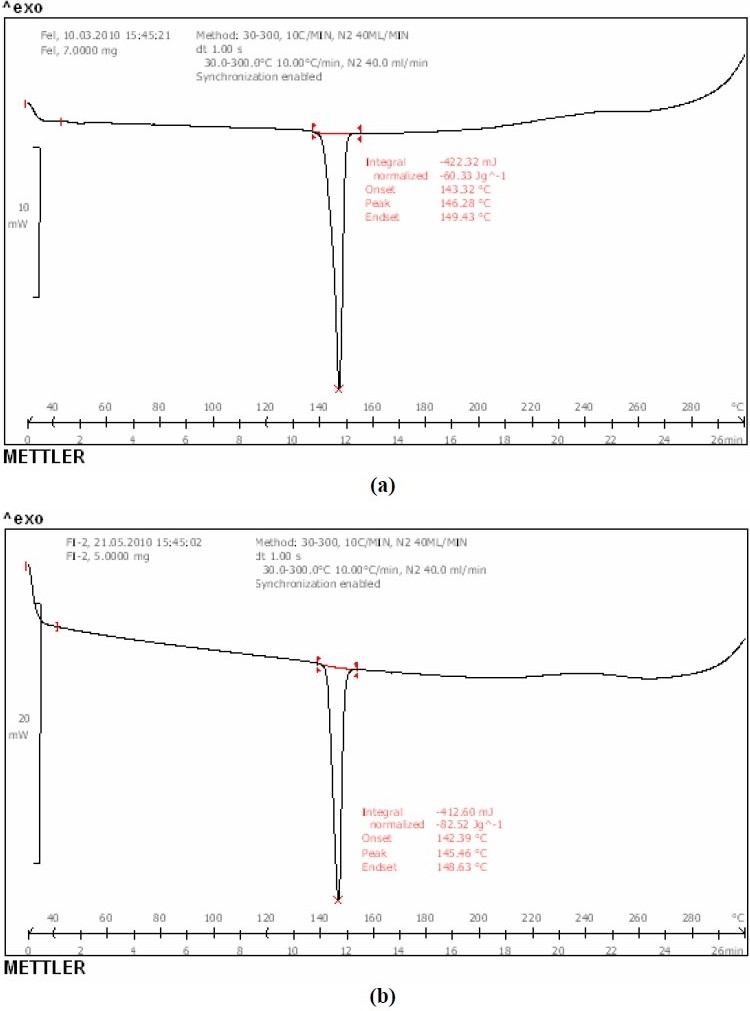
DSC Patterns of a) felodipine b) spherical agglomerates FI-2

**Fig. 5:**
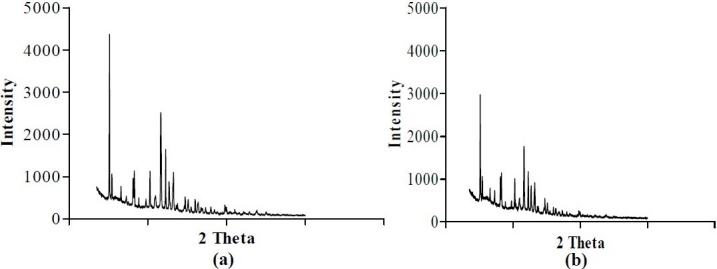
X-ray diffraction spectra a) felodipine b) spherical agglomerates FI-2

**Fig. 6:**
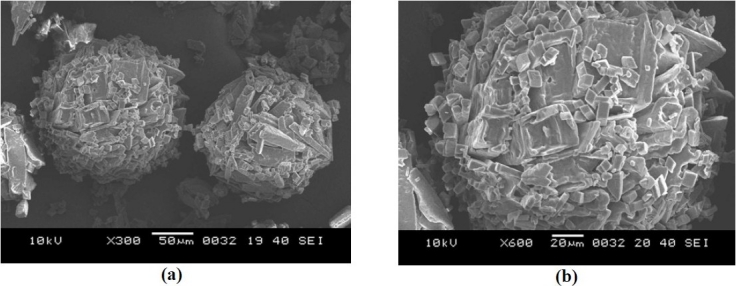
SEM micrographs of: a) spherical agglomerates FI-2 at ×300 b) spherical agglomerates FI-2 at ×600

**Fig. 7:**
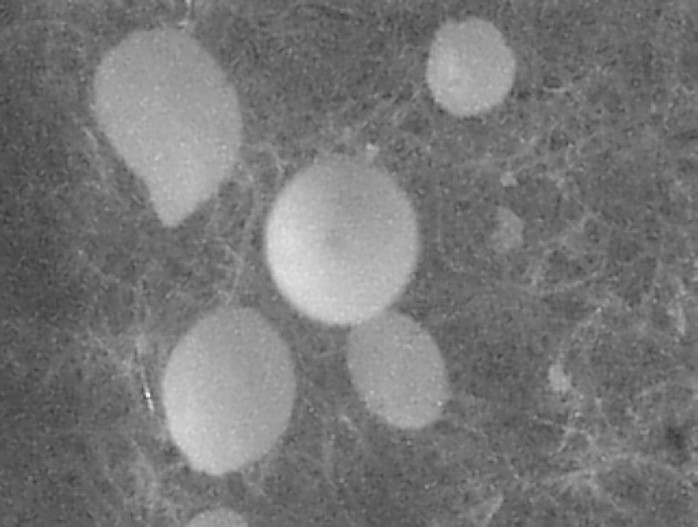
Optical micrograph of spherical agglomerates FI-2 at ×60

**Fig. 8:**
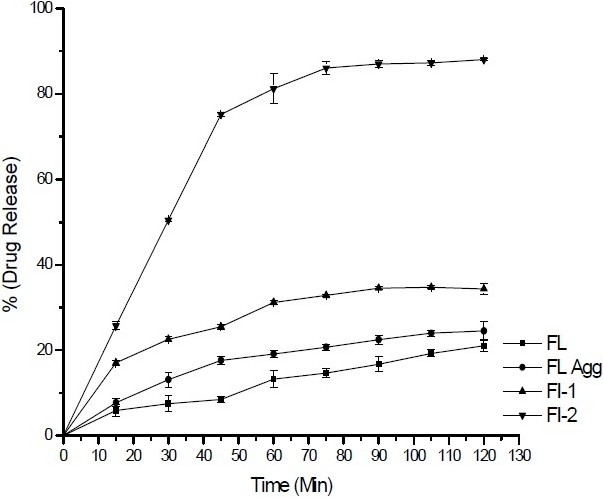
Dissolution profile of pure drug and agglomerates in Phosphate buffer 6.8 with 1.8% Tween 80. Mean ± SD, n=3

Table 1 shows the composition of spherical agglomerates with and without Inutec SP1. The percentage yield and drug loading efficiency is provided in Table 2. The yield of the recrystallized agglomerates was found to be between 91-94%, while drug loading efficiency was in the range of 94-100%. FTIR of FL as well as its spherical agglomerates is presented in Fig. 3. FTIR spectra of FL showed characteristic peaks at 3371.57 (N-H Str., Secondary), 2989.66 (C-H Str., -CH3), 3068.75 (C-H Str., Aromatic), 1689.64 (C=O Str.), 769.60 (C-Cl Str.) cm^-1^. There were no considerable changes in the IR peaks of the spherical agglomerates when compared to pure FL. The DSC thermograms of pure FL and its spherical agglomerates with Inutec SP1 are presented in Fig. 4. A single endotherm at 146.28 °C was ascribed to drug melting. There was a negligible change in the melting endotherms of prepared spherical agglomerates compared to pure drug (FI-2 = 145.46 °C). The results of PXRD of FL and spherical agglomerates are shown in Fig. 5. It shows presence of several distinct peaks in the PXRD of both pure drug powder and agglomerates of FL at a diffraction angle of 2θ 10.30°, 10.90°,16.30°, 16.58°, 20.56°, 23.32°, 24.56° and 26.54° which revealed that the pure drug and agglomerates were both in a crystalline form. Although the agglomerates showed crystalline pattern in PXRD diffractogram, but the intensity of the peaks have been reduced in comparison with the untreated powder. The results of surface morphology studies are shown in Fig. 6. The SEM results revealed the spherical structure of agglomerates. The pure drug exhibited a very small particle size (83.12 ± 10.21 µm, n=3) whereas the size of prepared agglomerates with Inutec SP1 was found to be 134.33 ± 13.57 µm, n=3 for FI-2. The shape of the crystals, when observed using an optical microscope was spherical in all the prepared agglomerated formulation (Fig. 7). Fig. 8 shows the results of dissolution profile of the untreated powder and spherical agglomerates of FL. The DE60 % of the agglomerates FI-2 was increased more than 4-fold compared to the untreated drug. As the figure indicates, FL was dissolved more than 85% from agglomerates after 120 min while the untreated powder was just dissolved about 20% at comparable time. The results revealed that the spherical agglomerates with 1% w/v Inutec SP1 (FI-2) caused significant increase (*P*<0.05) in drug release compared to the pure drug.

**Table 2:** Results of percentage yield and drug loading efficiency.

Formulation	Percentage yield	Drug loading efficiency
Felodipine (FL)	---	100.0 ± 0.00
FL Agg	93.23	100.0 ± 0.00
FI-1	92.61	97.54 ± 2.64
FI-2	91.08	94.18 ± 1.46

## DISCUSSION

Spherical agglomerates of FL were prepared by QESD using a three solvent system. It involves good solvent, poor solvent and a bridging liquid. The selection of these solvents depends on the miscibility of the solvents and the solubility of drug in individual solvent. Accordingly acetone, dichloromethane and water were selected as a good solvent, bridging liquid and poor solvent, respectively. FL is highly soluble in acetone, but poorly soluble in water. Also it is soluble in dichloromethane which is immiscible in water. Hence, this solvent system was used in the present study. In QESD method, when good solvent solution of drug plus bridging liquid were poured in the poor solvent (containing Inutec SP1) under agitation, quasi emulsion droplets of bridging liquid and good solvent were produced. Then the good solvent diffused gradually out of the emulsion droplet into the outer poor solvent phase. The counterdiffusion of the poor solvent into the droplets induces the crystallization of the drug within the droplet due to the decrease in solubility of the drug in the droplet containing the poor solvent. The bridging liquid wets the crystal surface to cause binding and promotes the formation of liquid bridges between the drug crystals to form spherical agglomerates. The spherically agglomerated crystals are formed by coalescence of these dispersed crystals. In FTIR study, there were no considerable changes in the IR peaks of the spherical agglomerates when compared to pure FL. The FTIR spectra of spherical agglomerates showed that no changes occurred in chemical structure and did not present a great fingerprint difference. DSC results further supported the IR spectroscopy results, which indicated the absence of any interactions between the drug and additives used in the preparation. However, there was a decrease, although very small, in the melting point of the drug in the spherical agglomerates compared to that of pure FL. This indicates the little amorphization of FL when prepared in the form of agglomerates. PXRD pattern showed distinctly a reduction in crystallinity percentage of the sample after reversion to spherical agglomerates. According to Bates and Ivanisevic(16), the percentage of crystallinity is directly related to the ratio between the integrated intensities caused by the crystalline and amorphous material in a similar measurement range of PXRD diffractogram. Furthermore, the result of PXRD indicates partial amorphization of the drug in its agglomerated form. The surface morphology studies also revealed that the agglomerates were formed by very small crystals, which were closely compacted into spherical form. These photomicrographs showed that the prepared agglomerates were spherical in shape. When the dissolved drug in acetone is added to the stirring water containing Inutec SP1, its solubility reduces and begins to precipitate. During the time and by evaporation of acetone a void space will be produced inside the aggregates. After complete evaporation of this good solvent and by simultaneous addition of dichloromethane the crystals will be wet and attached together due to the interfacial pressure and capillary forces, which is obvious in the micrographs. Enhancement in dissolution rate of spherical agglomerates as compared to pure drug may be due the presence of polymer, Inutec SP1, which is a polymeric surfactant with a high HLB value, hence its solubility in a lipophilic or a hydrophobic phase is extremely low. Due to the polymeric character, this type of surfactant tends to form aggregates when dispersed in water, resulting in a slightly turbid solution. However, this means that when an oil or hydrophobic particles are added to an aqueous dispersion of Inutec SP1, the polymeric surfactant will concentrate on the interface between the hydrophobic particles and water, leading to improved wetting and hence dissolution.

## CONCLUSION

The spherically agglomerates crystals of FL with Inutec SP1 were successfully prepared for enhanced dissolution rate properties of this drug by spherical crystallization technique. The micromeritic properties and also the dissolution profile of the drug were dramatically affected by this technique.
